# Geographic accessibility and hospital competition for emergency blood transfusion services in Bungoma, Western Kenya

**DOI:** 10.1186/s12942-023-00327-6

**Published:** 2023-03-27

**Authors:** Eda Mumo, Nathan O. Agutu, Angela K. Moturi, Anitah Cherono, Samuel K. Muchiri, Robert W. Snow, Victor A. Alegana

**Affiliations:** 1 Population Health Unit, Kenya Medical Research Institute-Wellcome Trust Research Programme, Nairobi, Kenya; 2 Department of Geomatic Engineering and Geospatial Information System (GEGIS), Jomo Kenyatta University of Agriculture and Technology (JKUAT), Nairobi, Kenya; 3 Centre for Tropical Medicine and Global Health, Nuffield Department of Medicine, University of Oxford, Oxford, UK

**Keywords:** Accessibility, Spatial competition, Blood transfusion, Travel time, Emergency, Bungoma

## Abstract

**Background:**

Estimating accessibility gaps to essential health interventions helps to allocate and prioritize health resources. Access to blood transfusion represents an important emergency health requirement. Here, we develop geo-spatial models of accessibility and competition to blood transfusion services in Bungoma County, Western Kenya.

**Methods:**

Hospitals providing blood transfusion services in Bungoma were identified from an up-dated geo-coded facility database. AccessMod was used to define care-seeker’s travel times to the nearest blood transfusion service. A spatial accessibility index for each enumeration area (EA) was defined using modelled travel time, population demand, and supply available at the hospital, assuming a uniform risk of emergency occurrence in the county. To identify populations marginalized from transfusion services, the number of people outside 1-h travel time and those residing in EAs with low accessibility indexes were computed at the sub-county level. Competition between the transfusing hospitals was estimated using a spatial competition index which provided a measure of the level of attractiveness of each hospital. To understand whether highly competitive facilities had better capacity for blood transfusion services, a correlation test between the computed competition metric and the blood units received and transfused at the hospital was done.

**Results:**

15 hospitals in Bungoma county provide transfusion services, however these are unevenly distributed across the sub-counties. Average travel time to a blood transfusion centre in the county was 33 min and 5% of the population resided outside 1-h travel time. Based on the accessibility index, 38% of the EAs were classified to have low accessibility, representing 34% of the population, with one sub-county having the highest marginalized population. The computed competition index showed that hospitals in the urban areas had a spatial competitive advantage over those in rural areas.

**Conclusion:**

The modelled spatial accessibility has provided an improved understanding of health care gaps essential for health planning. Hospital competition has been illustrated to have some degree of influence in provision of health services hence should be considered as a significant external factor impacting the delivery, and re-design of available services.

**Supplementary Information:**

The online version contains supplementary material available at 10.1186/s12942-023-00327-6.

## Background

Early and prompt access to emergency care services e.g., blood transfusion (BT), could potentially avert 54% of mortality among critical care patients in low- and middle-income countries (LMICs) [1]. Timely access to BT as an emergency healthcare intervention is a fundamental lifesaving procedure [2]. BT is necessary for a wide range of critical care conditions, including trauma, surgery, obstetric hemorrhage in women, severe anaemia resulting from malaria, and sepsis and for those who experience haematological crisis due to sickle cell disease and hemophilia. BT services are available at the apex of the health system, usually hospital levels. The demand for blood in these life-threatening conditions vary at spatial, age and gender dimensions.

Availability of safe replacement blood and transfusion services is poorer in LMICs, where demand is high compared to upper income countries [3]. International policies and guidelines have an emphasis on improving blood donation and clinical efficiency in the use of blood, storage and safety of blood products [4]. Far less guidance is provided on the optimized geographic accessibility (a measure of the burden of travel between the care-seeker’s location and the hospital) to blood by patients likely to require transfusion in LMICs.

There are many geographic and social determinants of accessibility to BT services covering both demand and supply factors. Demand-based factors include the geographic distance or travel time, perceived need to seek care influenced by cultural beliefs, and availability of transportation modes [5–7]. Physical distance to a transfusing health facility is one of the major reasons for the delay in receiving transfusion. Optimal travel times to health facilities vary depending on conditions, for example in maternal and neonatal care this is often defined as travel time within 2 h [8, 9], while for emergency and trauma related interventions this has been defined as within 1 h [10, 11]. Advances in availability of geographical data and spatial analysis techniques have improved how physical accessibility is defined, moving away from simplistic estimates of Euclidean distance. New methods include the dynamic inclusion of multimodal transportation and cost-surface analysis [12, 13]. These novel techniques depend on the availability of adequate data but have been used to improve our understanding of populations who intrinsically face access challenges due to longer travel distances [14].

Travel time has been investigated in several settings in sub Saharan Africa for severe conditions requiring timely hospital access, including obstetrics [15–19], very low-birth-weight infants [20], surgery [8], severe malaria [21] and other severe paediatric conditions [22]. These studies have largely focused on access to broad hospital, in-patient care but have not accounted for services available for specific conditions, including BT services. This could mainly be attributed to inadequate facility-level data on availability of different health services.

In Kenya, the World Bank estimates that seven people require a blood transfusion every 10 min, yet the country faces a shortage of supply, with only 16% of the blood needed in the country collected [23]. There is need to identify hospitals offering transfusion services and examine spatial competition between them to understand their degree of attractiveness based on geographically derived factors. Spatial competition between hospitals providing a health service such as BT can be associated with available supply at the facilities, available alternative hospitals and the lower-level facilities served by each hospital. The concept of competition (level of attractiveness) is well defined in social and economic disciplines such as the geography of education and banking services [24, 25]. However, the impact of hospital competition in the provision of BT and other key healthcare interventions in sub Saharan Africa (SSA) has not been defined [26]. Blood transfusion has been studied in Kenya in relation to pediatric clinical practice and outcomes [27–31] and initiatives to improve safety and management of blood [32]. Despite the expected high demand for blood, there have been no formal analyses of geographic accessibility and hospital competition in BT in Kenya.

The study aims to model the travel time to hospitals offering BT services in Bungoma county, Western Kenya as an exemplar of county level BT marginalization and assess spatial competition between these hospitals.

## Methods

### Blood provision context in Kenya

The Kenya National Blood Transfusion Service (KNBTS) was established in 2000 with support from US overseas development assistance. It supports blood donation drives, hemovigilance, blood storage and supply nationwide through six regional blood transfusion centres (RBTCs):^1^ Nairobi and Eastern region, Mount Kenya region, Lake region, North Rift region, Central Rift region, and Coast region and 45 satellite centres. Hospitals make requests for blood from the RBTCs which centrally manage collection, testing, processing, and distribution of blood. Distribution data is not publicly available from KNBTS. However, blood units ordered and received by health facilities are reported in the Kenya health information system (KHIS), that was launched in 2011. It is based on the district health information system version 2 (DHIS2) [33] which serves as a single, harmonised health reporting system for all surveillance programs in Kenya.

### Study site

**Fig. 1:**
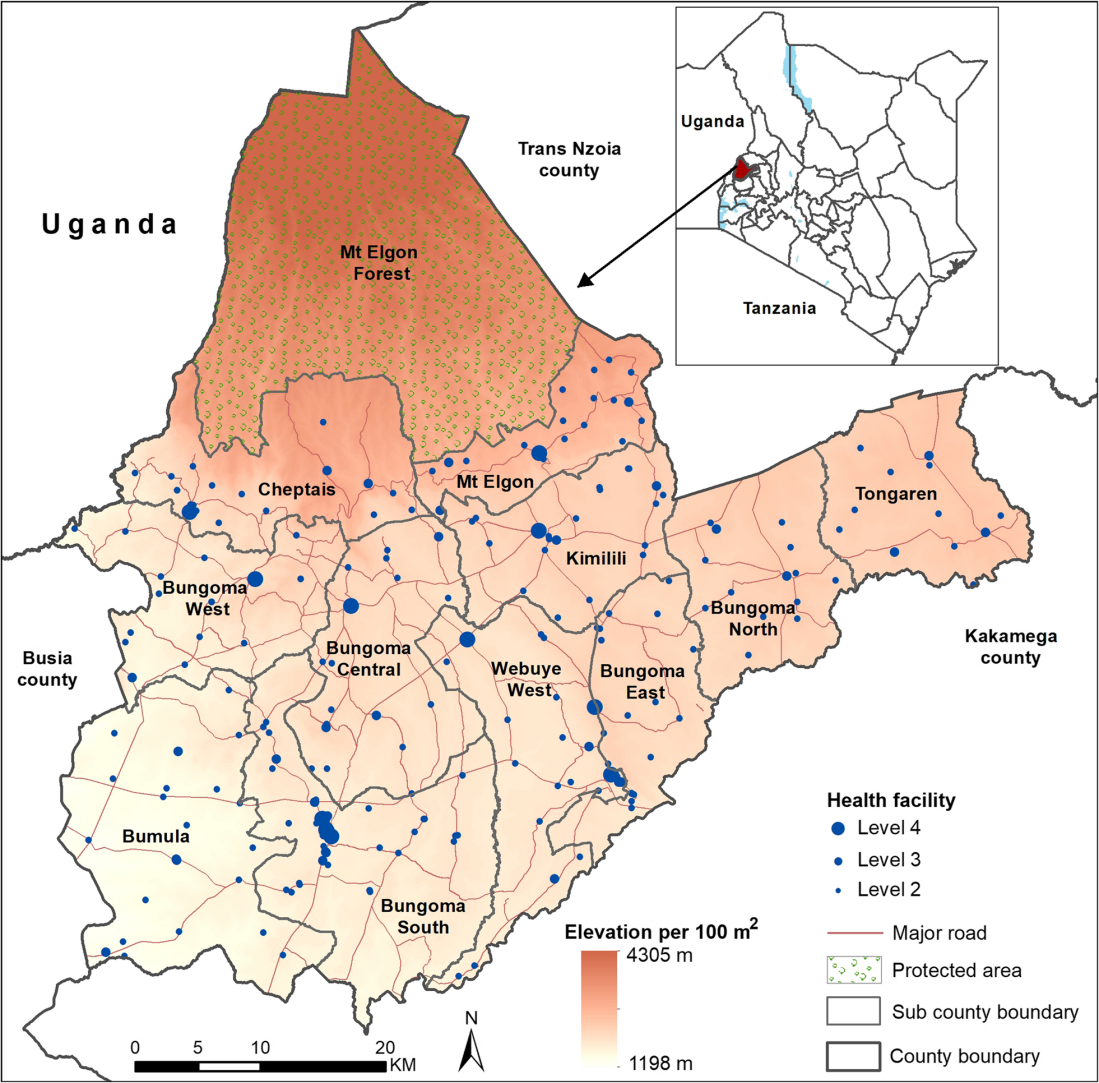
Spatial distribution of Bungoma’s health facilities from [42], the elevation as obtained from the advanced land observing satellite (ALOS) resampled to 10 m spatial resolution and the major roads as obtained from OpenStreetMap

Bungoma county is located in Western Kenya bordering Trans Nzoia, Kakamega, and Busia counties and shares a border with Uganda (Fig. 1). The county has a varied terrain from lowland areas of 1198 m above sea level (MASL) to peaks in the forested and protected areas of Mount Elgon (4305 MASL) (Fig. 1) which are key when defining access. It comprises 12 sub-counties, which serve as administrative sub-divisions for health planning [34], governed by the County Ministry of Health, located in Bungoma town, Bungoma South sub-county [35]. The health sector is configured according to national guidelines of service-level provision ranging from community-based services to fixed health facilities forming a referral pathway from level 2 to level 4/5 where essential emergency in-patient care services are organized [36]. Bungoma was selected because of intense, perennial levels of malaria transmission [37, 38], resulting in a significant demand for emergency care for severe malaria anaemia [39, 40] and an underlying high level of sickle cell homozygotes [41] that would require blood during crisis.

A total of 252 health service providers operate within the county, 143 (57%) managed by the local ministry of health, 27 (11%) by mission/NGO sectors and 82 (32%) private service providers [42]. These services are connected by 458 km of motorable road (Fig. 1).

### Defining a list of hospitals capable of providing blood transfusion services

All the health facilities in Bungoma county were available from an updated geocoded inventory of health facilities in Kenya [42] based on the Kenya master health facility list (KMHFL) and the KHIS. Facilities in Bungoma identified as level 2 in the inventory (n = 205) were removed as these are not expected to provide emergency care services [36] remaining with 47, level 3 and 4 health service providers.

Information on the annual number of blood units received from KNBTS and/or the Lake regional centre at Kisumu or satellite centre at Bungoma county hospital; and the number of units used for the years 2018, 2019, 2020 and 2021 were then extracted from KHIS for the higher-level facilities. The KHIS^2^ platform was accessed via the health information site for aggregate reporting and analysis with access rights provided by the Ministry of Health (MoH). A transfusing health facility was defined as a facility that had received and transfused at least one blood unit between 2018 and 2021. Reported units transfused at the facility included blood from KNBTS and blood donated by patient’s family members and friends. In addition, the number of inpatient beds in each transfusing facility and the total inpatient admissions from 2018 to 2021 were also extracted from KHIS as additional contextual information of the facilities.

### Spatial ancillary data to model travel time to transfusing health facilities

Ancillary datasets required in the accessibility model were assembled which included a detailed road network, land cover, a digital elevation model (DEM) and travel barriers. The detailed road network was obtained from OpenStreetMap^3^ and reclassification was done in ArcMap version 10.5 (ESRI Inc., Redlands, CA, USA) based on the designated road classes in the country; national, primary, secondary, minor, government, settlement, and rural roads. Land cover was obtained from the 2020 European Space Agency (ESA) world cover dataset^4^ available at 10 m spatial resolution with a 75% overall accuracy [43]. The study region had six land cover classes: tree cover areas, shrubland, bare land, grassland, built-up areas, and wetland which were used to inform travel impedances in regions that were not covered by the road network. A DEM from ALOS at 12.5 m spatial resolution was used to derive a slope-correction factor for each degree rise or drop in slope (Fig. 1). The DEM had been obtained from a radiometric terrain correction and resampling process of a 30 m DEM from Shuttle Radar Topographic Mission resulting to a superior product for use in scientific applications [44]. Additional travel barriers included were the major rivers and the protected areas as obtained from OpenStreetMap^5^ and the world database^6^ on protected areas respectively.

### Population data

Population datasets with fine spatial resolution are required to estimate served and marginalized populations. To overcome the lack of empirical high-resolution population data, we adopted the dasymetric spatial modelling approach developed by WorldPop [45]. It provides a 1 × 1 km surface of population density by redistributing census-based counts informed by remotely sensed data such as land cover, and night-time lights and shifts people from unlikely areas such as water bodies and assigns them to built-up areas. A scaling factor was determined by comparing the population in Bungoma from the 2019 WorldPop population density raster map^7^ with the 2019 census counts. The WorldPop 2019 population dataset was adjusted to match the census counts at the county level and projected to 2021 using the 2009–2019 intercensal growth rate [46]. The 2021 projected population raster was used in extracting population of interest in Bungoma using zonal statistics in ArcMap Version 10.5. Population was aggregated at the enumeration area (EA) level; an EA is a census unit that approximates to a village of circa 100 households [46].

### Modelling travel time to the transfusing health facilities in Bungoma

Travel time was modelled using the WHO AccessMod tool version 5 which uses a least-cost path algorithm (Dijkstra) to compute the fastest path between any spatial location and the nearest point (in our case a transfusing facility) [13]. A travel scenario combining walking followed by motorcycle and then vehicle was selected based on updated spatial accessibility literature [47]. The combined travel scenario assumed that a care-seeker/patient would first walk on the land cover in areas not covered by the road network, then use a motorcycle on the minor and special roads and take a vehicle to hospital along the higher-class roads. Travel speeds above each land cover and road class were assembled from previous comparable studies in Western Kenya [9, 48, 49] (Additional file 1: Table S1). The DEM was resampled to 10 m spatial resolution, and with all the other datasets: road network, travel barriers, and land cover (Additional file 1: Fig. S1) set to the same projected coordinate system in ArcGIS before importing them to AccessMod. In AccessMod, a merged land cover was first obtained by stacking the road network, travel barriers and land cover layers together. The “accessibility module” was then used to compute travel time to the transfusing health facilities using speeds from the defined travel scenario and the analysis was conducted at 10 m spatial resolution. Travel time was modelled for each of the transfusing facilities individually as well as for all the transfusing facilities collectively.

### Delineating catchment areas for the transfusing health facilities

Accessibility was further defined from the travel time results by defining service areas for the transfusing facilities assuming a uniform risk of emergency occurrence across the county. A 3-step floating catchment area (3-SFCA) method was used which assumed that the demand for health services from the population was influenced by availability of alternative health facilities within reach [50]. The method was implemented in R using the *fca package* [51] with 3 key input datasets: demand (P_i_), supply (S_j_) and a travel time matrix. Demand was represented by population extracted to each EA in the county and the EA centroids were generated to represent the demand locations. The supply dataset comprised the general capacity of the transfusing facilities defined by the total inpatient admissions from 2018 to 2021 as reported in KHIS. The travel time matrix was generated for each demand and supply pair resulting to the travel time from each EA centroid to each of the transfusing facilities as extracted in ArcGIS Version 10.5.

The first step in the 3-SFCA was to generate a selection weight (G_ij_) from the travel time matrix which defined the probability of population at an EA (i) to seek health services at a transfusing facility (j). This probability was influenced by the travel time between i and j and the travel time from i to all other transfusing facilities. It allowed for accounting of alternative transfusing facilities available to population in a specific EA. A travel time weight (W_r_) was computed from the travel time between i and j considering all travel time weights of other transfusing facilities in the pre-defined 1-h catchment. The weighting was done gradually with less weights being assigned with increasing travel time. The assigning of weights followed a gaussian decay function which quantified the extent that selection weight improved if a transfusing hospital was only 5 min away compared to the hospital being at the selected maximum threshold of 60 min. The gaussian decay function used was:

$${\varvec{f}}({\varvec{t}})={{\varvec{e}}}^{\frac{-({{\varvec{t}}}^{2})}{{\varvec{\beta}}}}$$

where ***t*** denotes the travel time and ***β*** the friction coefficient which determined the magnitude of the travel time decay function [52, 53].

The selection weight (${\varvec{G}}_{\varvec{ij}}$) was then computed as:

$${\varvec{G}}_{\varvec{ij}}=\frac{{\varvec{W}}_{\varvec{r}}}{\sum_{{\varvec{j}}\in ({\varvec{T}}_{\varvec{ij}}\le {\varvec{T}}_{\varvec{max}})}{\varvec{W}}_{\varvec{r}}}$$

where ${\varvec{G}}_{\varvec{ij}}$ is the probability that population ${\varvec{i}}$ selects facility ${\varvec{j}}$, ${\varvec{W}}_{\varvec{r}}$ the travel time weight, ${\varvec{T}}_{\varvec{ij}}$ travel time between ${\varvec{i}}$ and ${\varvec{j}}$ and ${\varvec{T}}_{\varvec{max}}$ is the maximum travel time threshold of 60 min.

The second step used the selection weight to calculate the supply–demand ratio (${\varvec{R}}_{\varvec{j}}$) which distributed the demand from a specific EA to several transfusing facilities based on the probability of selection. This ratio was calculated as:

$${\varvec{R}}_{\varvec{j}}=\frac{{{\varvec{S}}}_{\varvec{j}}}{\sum_{{\varvec{i}}\in ({\varvec{T}}_{\varvec{ij}}\le {\varvec{T}}_{\varvec{max}})}{\varvec{G}}_{\varvec{ij}}{\varvec{P}}_{\varvec{i}}{\varvec{W}}_{\varvec{r}}}$$

where ${\varvec{S}}_{\varvec{j}}$ is the capacity of transfusing facility ${\varvec{j}}$ and ${\varvec{P}}_{\varvec{i}}$ is the population at EA ${\varvec{i}}$.

In the last step, the spatial accessibility index (SPAI) was computed for each EA as:

$${\varvec{SPAI}}_{\varvec{i}}=\sum\limits_{{\varvec{T}}_{\varvec{ij}}\le {\varvec{T}}_{\varvec{max}}}{{\varvec{G}}}_{\varvec{ij}}{\varvec{R}}_{\varvec{j}}{{\varvec{W}}}_{\varvec{r}}$$

Obtained SPAI values were interpreted and classified appropriately based on their quintiles to get EAs in low, medium, and high spatial accessibility levels.

Consequently, the modelled SPAI metrics were used to calculate the mean SPAI for each sub-county. The projected population grid and the classified SPAI levels were used to estimate the percentage of people per sub-county residing in the 3 accessibility classes in ArcMap version 10.5 (ESRI Inc., Redlands, CA, USA). A comparison of the marginalized population derived from the travel time and SPAI results was conducted to assess output agreement.

### Spatial competition (attractiveness) between transfusing hospitals

The degree of spatial competition in the county healthcare market was defined within facility catchment constrained to 1-h travel time, optimal for emergency BT patients [54]. Spatial competition index (SCI) was computed for each facility to represent the level of service demand and availability of alternative facilities within the defined facility catchment. That’s how attractive a transfusing facility is to those in need of blood transfusion services. Lower-level, non-transfusing facilities in a facility catchment were used to estimate BT demand as they dictate flow of patients through referrals to the transfusing facilities. SCI was computed as:

$${\varvec{SCI}}_{{\varvec{i}}}=\frac{1}{{{\varvec{n}}}_{\varvec{i}}}{\sum }_{1}^{{\varvec{n}}_{\varvec{i}}}\frac{1}{{\varvec{k}}_{\varvec{i}}}({\varvec{W}}_{\varvec{i}})$$

where ${\varvec{i}}$ is the transfusing facility with ${\varvec{n}}$ low-level facilities and ${\varvec{k}}$ alternative transfusing facilities within its 1-h travel time catchment while ${\varvec{W}}$ is the weighting factor.

The weighting factor accounted for the relative size of each transfusing facility and was calculated by dividing the number of available inpatient beds at each hospital by the total inpatient beds available at all the transfusing hospitals.

For a comparison with the computed competition metric, the market share or the relative contribution of each facility to providing blood transfusion services over the 4 years, was also computed.

### Validation of the competition index

Finally, to understand whether highly competitive facilities had better capacity for blood transfusion services we assessed the relationship between the facilities’ BT capacity and the computed SCIs. As representative variables for BT capacity, we used blood units transfused in the hospital and blood units obtained from KNBTS. The relationship was based on Pearson correlation, the choice which was informed by quantile–quantile (Q–Q) plots that explore the distribution of the three variables under consideration.

## Results

### Overview of blood transfusion sites and volumes

**Fig. 2:**
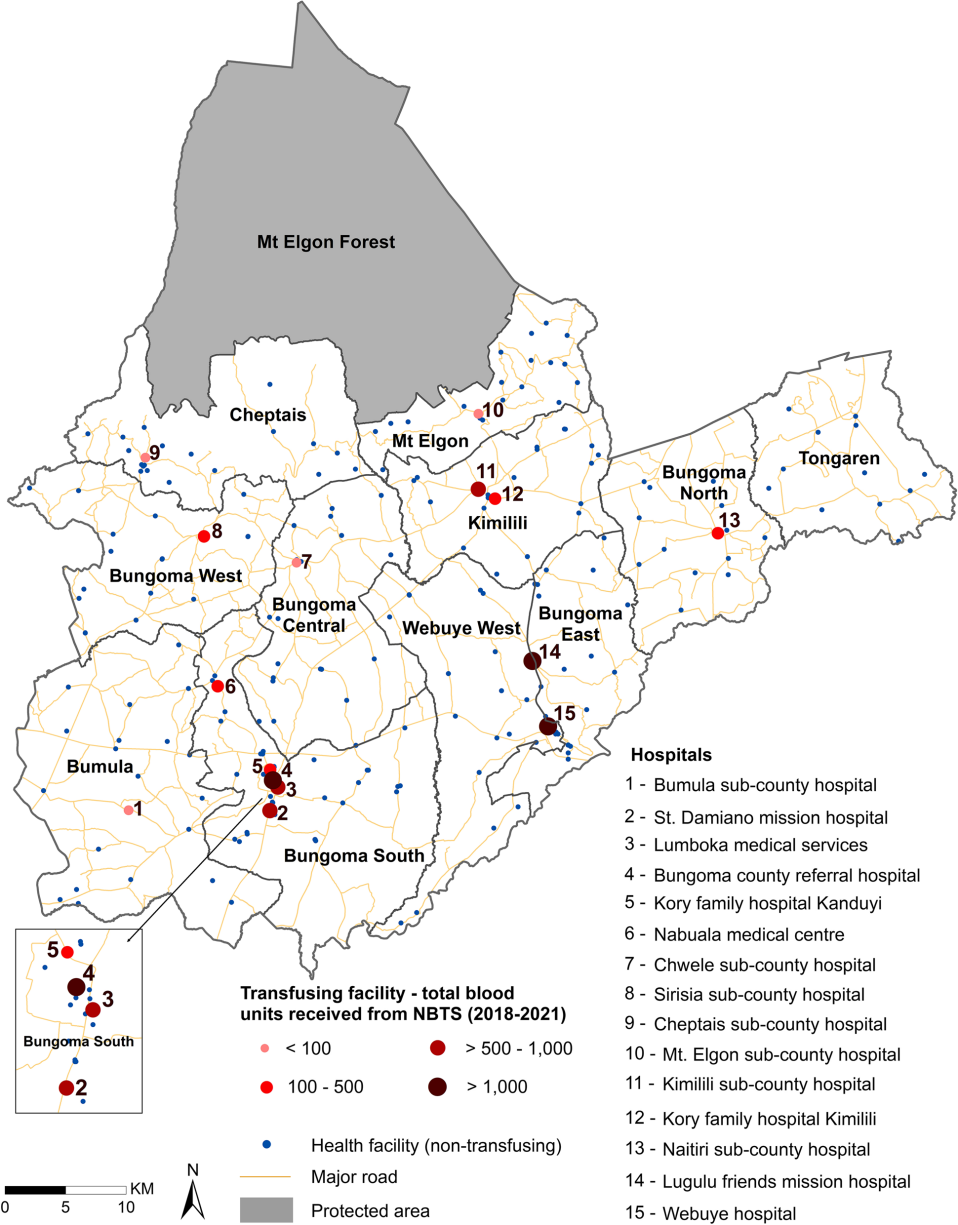
Spatial distribution of the 15 transfusing facilities in the county with the dot size varied based on the total number of blood units received by each facility from NBTS from 2018 to 2021

There were 252 health facilities in Bungoma county and 15 of these facilities received or provided at least one blood unit between 2018 and 2021 from the NBTS or regional/satellite centres. These included the level 4 county referral hospital at Bungoma town, eight level 3 sub-county hospitals managed by the MoH, two level 3 mission hospitals and four level 3 private-for-profit hospitals. The 15 hospitals were distributed among the sub-counties as follows: Bungoma South/Kanduyi (n = 5), Kimilili (n = 2) and Webuye West (n = 2). Each of the other sub-counties had one transfusing facility except for Tongaren, which lacked a transfusing facility (Fig. 2).

A total of 16,662 units of blood were received from KNBTS and 22,843 units used (including replacement donors at the facility) for transfusion at these 15 facilities over 4 years 2018–2020 (Table 1). The average per annum receipt and use of blood units per facility ranged from 10 to > 1000 and 12 to > 1000 respectively. The highest volume of blood, 13,679 units (Table 1) was transfused at Bungoma County Hospital.

**Table 1:** Characteristics of transfusing facilities: number of inpatient beds, competing and low-level facilities, total blood units from 2018 to 2021 and the computed competition metrics

Transfusing facility	No. of inpatient beds	No. of facilities in the 1-h catchment		Blood units (2018–2021)		Competition	
		Competing transfusing facilities	Low level facilities (non-transfusing)	Received from NBTS	Transfused at facility	Market share (%)	SCI
Bungoma County Referral	211	9	144	9543	13,679	16.31	0.21
Webuye Hospital	224	11	169	2888	4098	14.17	0.18
Kimilili Subcounty Hospital	81	5	117	503	809	11.27	0.14
Kory Family Hospital Kimilili	75	5	117	244	311	10.44	0.11
Cheptais Sub County Hospital	34	3	52	20	24	7.89	0.10
Lugulu Friends Mission	122	12	168	1135	1254	7.07	0.09
Naitiri Sub County Hospital	40	5	98	229	271	5.57	0.07
Mt Elgon Sub County Hospital	39	5	68	63	49	5.43	0.07
Bumula Sub County Hospital	37	6	85	58	70	4.29	0.06
Nabuala Medical Centre	65	11	149	131	241	4.11	0.05
St Damiano Mission	68	12	161	600	569	3.94	0.05
Sirisia Sub County Hospital	31	6	92	147	138	3.6	0.04
Chwele Sub County Hospital	41	10	115	53	39	2.85	0.03
Lumboka Medical	27	10	159	867	1101	1.88	0.02
Kory Family Hospital Kanduyi	17	10	166	181	190	1.18	0.01

From the correlation test, there was a significant positive association between the competition index and the hospital's transfusion capacity variables, specifically, the number of blood units received at the hospital (t = 3.57, p = 0.003, Pearson’s r = 0.70, n = 15, 95% CI [0.29, 0.89]) and the number of blood units transfused (t = 3.66, p = 0.002, Pearson’s r = 0.71, n = 15, 95% CI [0.31, 0.89])

### Physical access to the transfusing hospitals and their delineated catchments

**Fig. 3:**
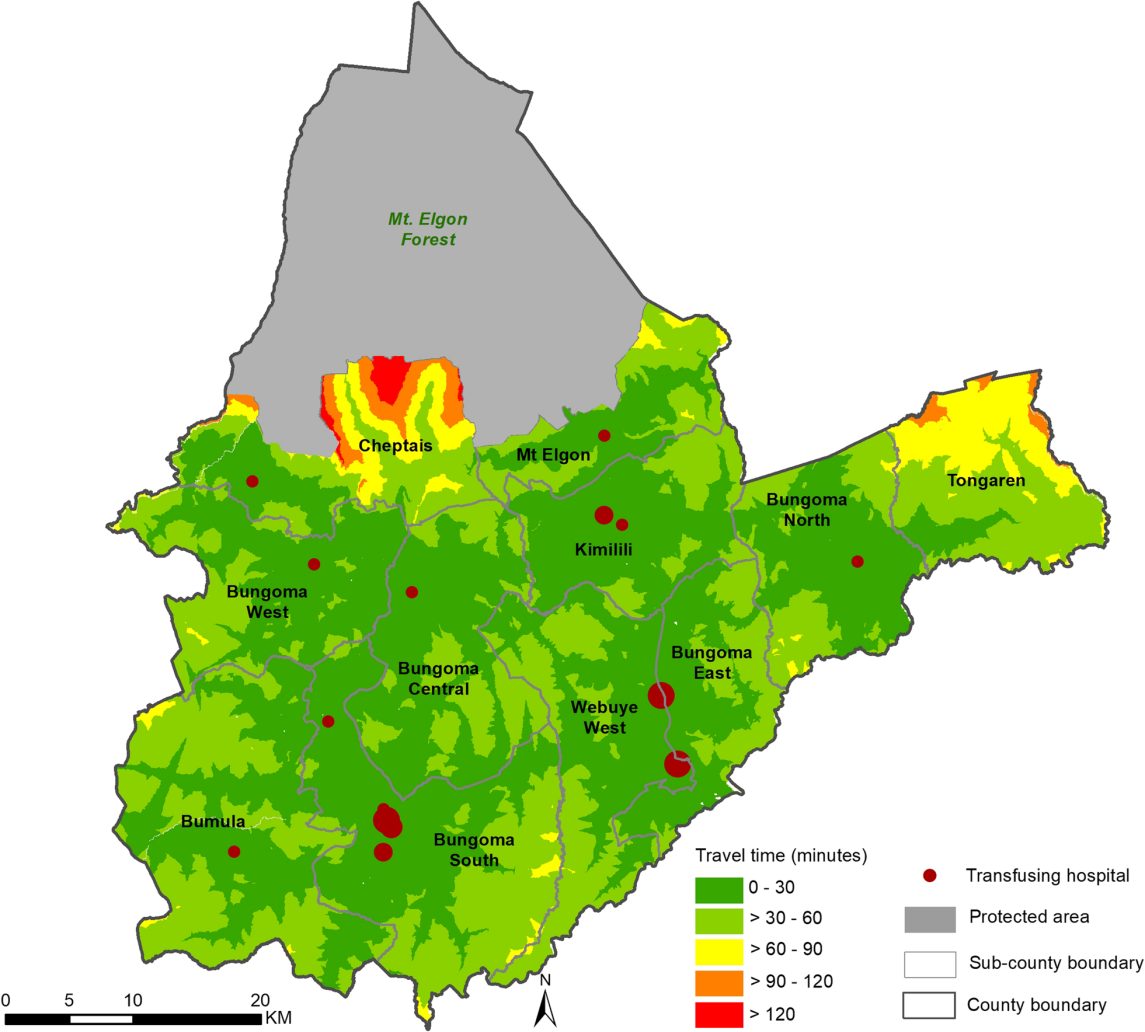
Hospitals accessibility based on travel time from defined travel scenario where dark green represents areas within 30 min of a transfusing hospital and the medium green show areas more than 30 min but less than 1 h to the nearest hospital

The average travel time from any population location in the county to the transfusing facilities (n = 15) was 33 min based on the collectively modelled travel time but this varied by subcounty (Fig. 3). Bungoma Central subcounty had the lowest average travel time of 25 min while Tongaren had the highest average travel time of 58 min.

All sub-counties, except Tongaren and Cheptais, had over 90% of their population residing within the 1-h catchment with Bungoma Central having almost all its population, 99.9%, living within the catchment. Tongaren had the largest percentage, 33.5%, of marginalized people from the 1 h travel time catchment, followed by Cheptais with 27.8% (Additional file 1: Table S2). At the county-level, 1.6 million people (95.1%) lived within the 1-h travel time catchment of a transfusing facility out of which 1.1 million (60.9%) lived within 30 min of a transfusing facility. 85,056 people (4.9%) lived outside the 1-h travel time catchment and were therefore deemed marginalized.

**Fig. 4:**
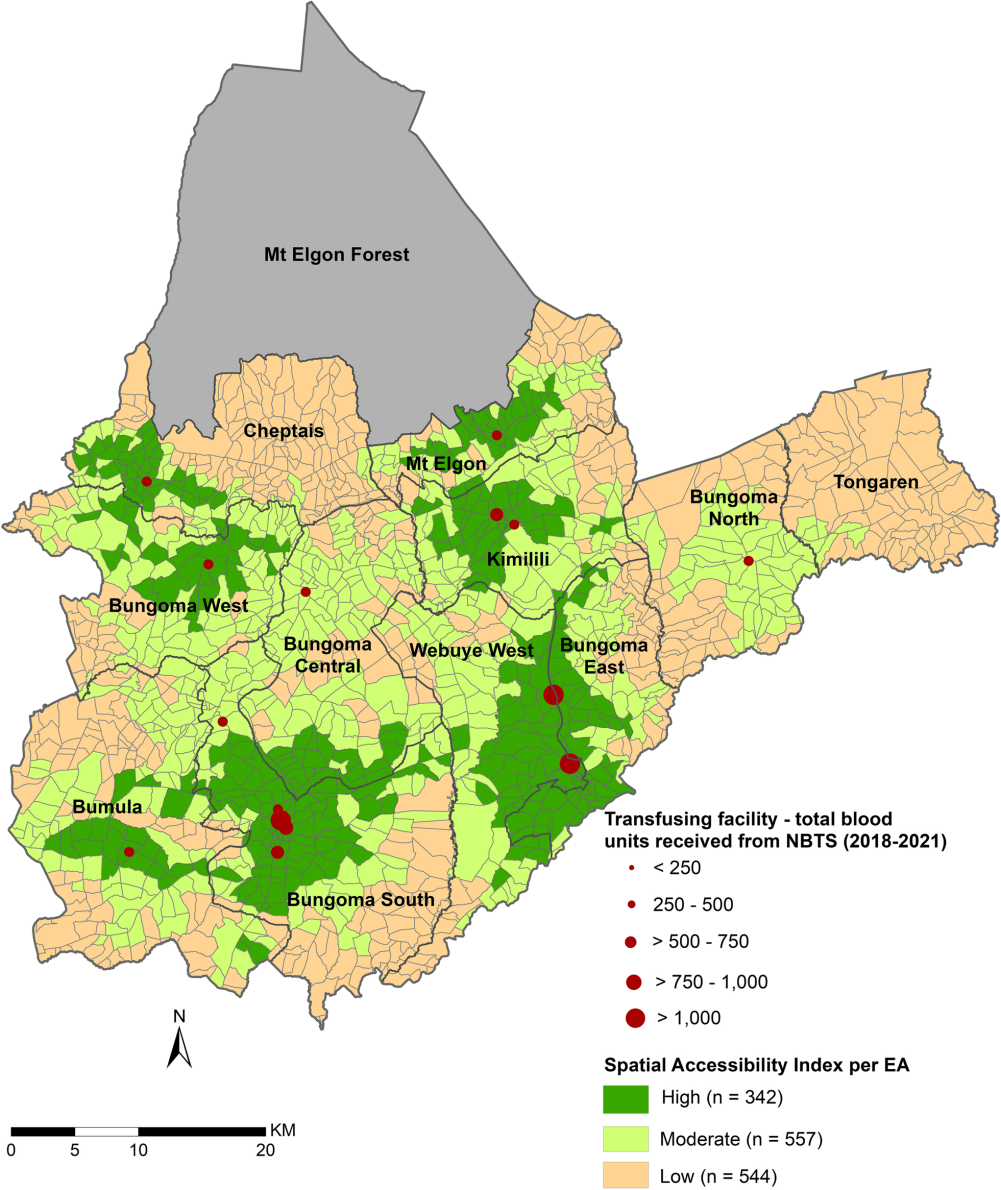
Spatial accessibility index per enumeration area estimated using the 3-SFCA method, aggregated into 3 accessibility level classes across the county: high, moderate and low accessibility EAs

Further assessment of spatial accessibility metrics at the EA level using the 3-SFCA resulted in SPAI values that ranged from 0 to 5.7. Three levels of spatial accessibility were created by aggregating the SPAI values and the number of EAs in each level were extracted: > 1.5—high accessibility with 342 EAs, > 0.5–1.5—moderate accessibility with 557 EAs and ≤ 0.5—low accessibility with 544 EAs (Fig. 4). The county’s headquarter, Bungoma South/Kanduyi subcounty, had 156,203 people (51.6%) living in the high accessibility class, while Bungoma North and Tongaren sub counties had no residents in this class (Additional file 1: Table S3). Bungoma Central had the highest population (114,937 people—64.1%) in the moderate accessibility level. Tongaren had the highest percentage of its population (93.2%) in the low accessibility class, followed by Cheptais subcounty with 56.4%. Overall, 27.9% (483,801 people) of the county’s population resided in the high accessibility EAs while 38.5% (668,973 people) and 33.6% (582,126 people) lived in the moderate and low spatial accessibility class respectively.

### Estimated spatial competition between the transfusing facilities

Table 1 shows the computed competition metrics and the variables used. In the 1-h travel time catchment, computed SCI ranged from 0.01 for non-competitive transfusing hospitals to 0.21 for the most competitive. Bungoma county and referral hospital was the most competitive facility with a share of 16% in the health market (Table 1). Webuye hospital, Kimilili subcounty hospital and Kory family hospital—Kimilili were also significantly competitive with SCI > 0.1 and a market share > 10%. Webuye Hospital was less competitive than Bungoma county hospital even though it had a larger capacity with 13 more beds. Facilities sharing a catchment area such as Kimilili subcounty hospital and Kory family hospital—Kimilili had their SCI metrics varied by their sizes as defined by their number of inpatient beds. Kory family hospital in Kanduyi/Bungoma South was the least competitive despite being in a sub county with relatively good spatial accessibility levels as depicted in Fig. 4.

## Discussion

The study analysed spatial accessibility to identified transfusing facilities in Bungoma at a fine spatial resolution and estimated spatial competition between these facilities. 95% of the county’s residents had timely access to the nearest identified transfusing hospital, although some vulnerable people would have to travel further than the recommended one-hour. Only 6% of the county's facilities were transfusing facilities, which were unevenly distributed among the sub-counties. Regions without a transfusing facility were considered to be the most marginalized with accessibility disparities further highlighted at the sub county level. Travel time and SPAI gridded surfaces were overlaid with the population raster to estimate the marginalized population which showed more variation in access levels across the sub-counties.

High accessibility in Bungoma Central was consistent with findings from [9], where the Central region has an effective, current transport network. Majority of the roads in this network are national trunk, primary, and secondary roads, all of which have higher speed limits. Tongaren sub-county was a poor accessibility area resulting from lack of a transfusing facility and its rugged terrain. The five hills in Tongaren: Bunambo, Sikuku, Sikulu, Milimani, and Matukhuli [55], constitute the steep slopes that affect all transportation modes, making the region difficult to access. Findings from the sub county level disparities highlight on the need for the county government and the private sector to equip more hospitals with transfusion capacities prioritizing Tongaren and Cheptais sub county.

SPAI delineation produced a higher proportion of underserved areas and marginalized populations than those derived from the travel time surface. This demonstrated that, as has previously been seen in other studies, the use of travel time measurements alone, without considering other relevant aspects like the demand and supply for health services, leads to an overestimation of spatial accessibility [56]. The non-constant demand model used in SPAI delineation, modifies a population point's demand by a certain amount depending on the available supply hence accounting for the overestimation [51]. Previous studies have emphasized the importance of additional factors, such as competition, the care-seeker’s socio-economic status and facility preference, which influence access to a facility [7, 57].

From the competition modelling findings, four transfusing hospitals located in Bungoma, Webuye, and Kimilili town were the most competitive. This showed that facilities in urban areas had a spatial competitive advantage over those in rural areas. This is due to improved spatial accessibility in urban regions [58], which provide for alternative competing facilities within reach in urban facility catchments. In addition, facilities in urban areas have bigger capacities in provision of healthcare, mostly due to the large volume of patients they serve but also because they are prioritized by the county government [59]. This depicts inequalities in healthcare access within the county as residents in rural areas must travel long distances to a facility since there are no nearby alternatives and while there, the required health service or product e.g., blood might not be adequate. Despite being a comparably larger facility based on the number of inpatient beds, Webuye hospital was less competitive than Bungoma county hospital. This can be attributed to the fact that Webuye Hospital needs to serve more low-level, non-transfusing facilities within its catchment than Bungoma County Hospital. The competition metrics highlight the need to establish additional transfusing facilities especially in catchments with low competition facilities to improve geographic accessibility and availability of blood transfusion services while also providing for alternative competing facilities.

The study also set out to evaluate the relationship between competition and the provision of blood. Findings indicated that the most competitive facilities receive and provide more blood units. Although it is rarely considered when establishing transfusion centres, spatial competition is potentially a driver in blood provision. Highly competitive facilities have also been linked to improved clinical outcomes for adult congenital heart disorders in [54], highlighting the importance of taking spatial competition into account in future research. However, our study used a small sample size of transfusing facilities hence future research could explore spatial competition with an increased sample size preferably a nation-wide coverage.

## Study limitation

A limitation in the travel time modelling process which could have affected the results was that delays due to traffic and seasonal variations affecting transport such as rains were not considered. Furthermore, use of EA centroids to represent the demand location is to some extent inaccurate because the centroid is only one spatial location which is misrepresentative of the people’s actual household locations.

We did not examine the efficacy, safety, or quality of the usage of blood at the 15 transfusing facilities. These are significant factors [60, 61] that are outside the scope of this study and should be taken into account in future work.

## Conclusion

We have defined spatial accessibility to transfusing health facilities based on simple travel time metrics and an improved floating catchment area estimation. The findings suggest that use of the simple travel time metrics may result to an overestimation of physical accessibility to health care which could mis-inform health committees and ministries in their planning process. Our study supports the need for using an improved gravity-based spatial access model to account for significant factors such as supply of health services and demand from the catchment population. The county government can utilize the improved spatial accessibility results when planning for allocation of health resources.

Spatial competition has been illustrated to have some degree of influence in provision of health services. Although our results merit further validation, spatial competition should be considered as a significant external factor impacting the delivery of health services. From the competition findings, policymakers can target hospitals with weak competition hospitals by enhancing their service delivery and infrastructure. This will lead to their increased utilization, which would alleviate overcrowding in the highly competitive hospitals. This study significantly adds to the already scarce spatial competition literature by providing the first assessment of the relationship between spatial competition and blood transfusion. It also lays foundation for future spatial competition studies that might include other important health care interventions and services.

## Supplementary Information

**Additional file 1.** A summary of the travel speeds selection, secondary datasets used in accessibility modelling, study's conceptual framework and the population extracted at each of the accessibility levels.

## Data Availability

Aggregated DHIS2 data is available online in the KHIS platform with access provided by Ministry of Health https://hiskenya.org/dhis-web-commons/security/login.action. The datasets used during this current study are also available from the corresponding author on reasonable request.

## Abbreviations

BT: Blood transfusion
LMICs: Low and Middle Income Countries
SSA: Sub-Saharan Africa
KNBTS: Kenya National Blood Transfusion Service
RBTCs: Regional blood transfusion centres
KHIS: Kenya health information system
DHIS2: District health information systems version 2
MASL: Meters above sea level
NGO: Non-Governmental Organization
ALOS: Advanced land observing satellite
KMHFL: Kenya master health facility list
DEM: Digital elevation model
ESA: European Space Agency
EA: Enumeration area
WHO: World Health Organization
3-SFCA: 3-Step floating catchment area
SPAI: Spatial accessibility index
SCI: Spatial competition index
MOH: Ministry of Health

## Footnotes

1 https://nbtskenya.or.ke/, accessed September 25, 2022.

2 https://hiskenya.org/dhis-web-commons/security/login.action, accessed July 10, 2022.

3 https://data.humdata.org/dataset/hotosm_ken_roads, accessed July 15, 2022.

4 https://worldcover2020.esa.int/viewer, accessed July 15, 2022.

5 https://data.humdata.org/dataset/hotosm_ken_waterways, accessed July 15, 2022.

6 https://www.protectedplanet.net/en, accessed July 15, 2022.

7 https://www.worldpop.org/datacatalog/, accessed July 19, 2022.
