# Supplementary material for: Geographic accessibility and hospital competition for emergency blood transfusion services in Bungoma, Western Kenya
Source: Int J Health Geogr. 2023 Mar 27;22:6. doi: 10.1186/s12942-023-00327-6 (PMC10041813; doi:10.1186/s12942-023-00327-6)
Supplement: Supplementary file 1 — Additional file 1. A summary of the travel speeds selection, secondary datasets used in accessibility modelling, study's conceptual framework and the population extracted at each of the accessibility levels. [file 12942_2023_327_MOESM1_ESM.docx]

**Geographic accessibility and hospital competition for emergency blood transfusion services in Bungoma, Western Kenya**

Eda Mumo^1,2*^, Nathan O Agutu^2^, Angela K Moturi^1^, Anitah Cherono^1^, Samuel K Muchiri^1^, Robert W Snow ^1, 3^, Victor A Alegana^1^

^1^ Population Health Unit, Kenya Medical Research Institute-Wellcome Trust Research Programme, Nairobi, Kenya

^2^ Department of Geomatic Engineering and Geospatial Information System (GEGIS), Jomo Kenyatta University of Agriculture and Technology (JKUAT), Nairobi, Kenya

^3^ Centre for Tropical Medicine and Global Health, Nuffield Department of Medicine, University of Oxford, Oxford, United Kingdom

* **Corresponding author**

Email

EM: [eddahwangui84@gmail.com](mailto:eddahwangui84@gmail.com)

NOA: [nagutu@jkuat.ac.ke](mailto:nagutu@jkuat.ac.ke)

AKM: [amoturi@kemri-wellcome.org](mailto:amoturi@kemri-wellcome.org)

AC: [acherono@kemri-wellcome.org](mailto:acherono@kemri-wellcome.org)

SKM: [smuchiri@kemri-wellcome.org](mailto:smuchiri@kemri-wellcome.org)

RWS: [rsnow@kemri-wellcome.org](mailto:rsnow@kemri-wellcome.org)

VAA: [aleganav@who.int](mailto:aleganav@who.int)

# **Additional information**

### **Travel speeds selection**

Modelling travel time in *AccessMod* requires definition of travelling speeds in km/hr. of the various transportation modes in the selected transport scenario [1]. A combined travel scenario was used in this study and speeds were assigned to each road class and land cover type (Table S1). The adopted speeds were assembled from comparable healthcare spatial accessibility studies done in Western Kenya [2-4].

**Table S1**: Travel speeds adopted in the modelling of travel time to the health facilities offering blood transfusion services using a combined travel scenario (walking followed by a motorcycle then a vehicle).

| Road classes | Speed in Km/hr. | Travel scenario |
| --- | --- | --- |
| National roads | 65.0 | Vehicle (Motorized) |
| Primary and secondary roads | 50.0 | Vehicle (Motorized) |
| Minor roads | 30.0 | Motorcycle (Motorized) |
| Government roads | 20.0 | Motorcycle (Motorized) |
| Settlement roads | 20.0 | Motorcycle (Motorized) |
| Rural and unclassified roads | 20.0 | Motorcycle (Motorized) |
| Land cover type |  |  |
| Tree cover areas | 2.5 | Walking |
| Shrubland | 5.0 | Walking |
| Bare land/ sparse vegetation | 5.0 | Walking |
| Grassland | 3.5 | Walking |
| Built-up areas | 5.0 | Walking |
| Wetland | 0.0 | Walking |

### **Study site and secondary datasets used for modelling travel time**

Bungoma county is one of the 47 counties formed after the establishment of Kenya’s devolved system of governance in 2013. The total land mass of the county is 3024 Km^2^ with only 2418 Km^2^ populated; in 2019 it was estimated that there were 1.67 million residents [5]. Under five mortality is high, 65 per 1000 live births [6] and has an absolute poverty index of 30% ranking 29 out of the 47 counties [7].

Ancillary datasets included factors that affect a care-seeker’s journey to the health facility. These comprised of the existing road network, rivers, protected areas, and the land cover (Figure S1).


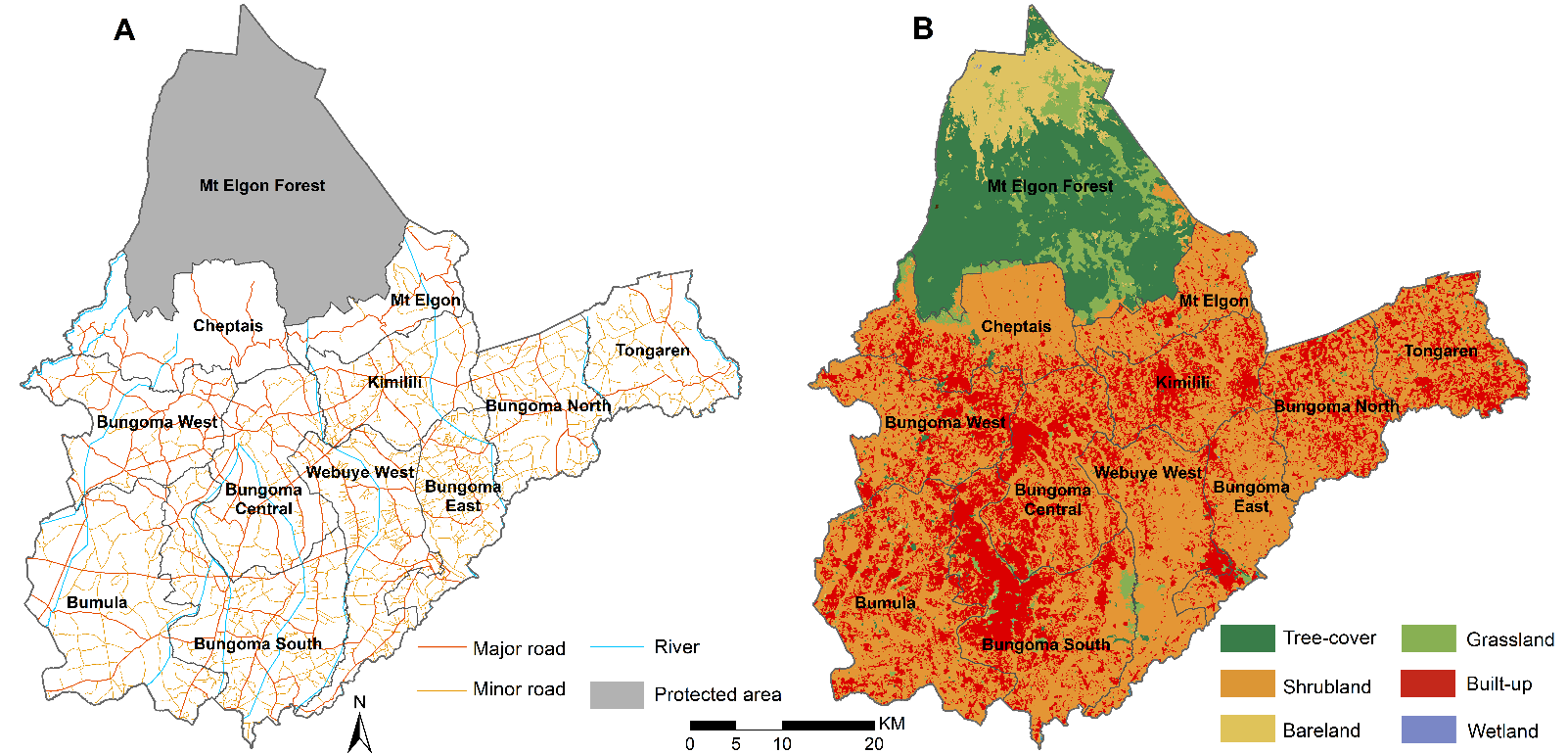


**Figure S1**: Assembled ancillary datasets for travel time modelling. (A) Detailed road network from OpenStreetMap and the travel barriers (rivers from OpenStreetMap and protected areas from the world database of protected areas); (B) land use land cover (LULC) surface in 2020 obtained from the European Space Agency (ESA) world cover dataset.

### **Methodology**

The modelling process for obtaining the spatial accessibility and competition metrics is summarized in figure S2. It includes the key datasets used in each process and the resulting outputs as well.

Supply – Hospital capacity

Demand – Population per EA

Spatial accessibility index

Low-level health facilities

Competition metrics

Hospital – No. of inpatient beds

Transfusing hospitals

Land cover, roads, DEM, rivers, and protected areas

Travel time

Blood units – received and transfused

Outcome/ Relationship

**Legend**

Input data

Modelling process

Output

**Figure S2**: The analytical process adopted in the study showing the four main modelling techniques used in obtaining the results.

### **Summary of extracted population in different accessibility levels**

**Table S2**: Mean travel time in each subcounty and the population residing in different travel time thresholds (within 30 minutes and 1 hour) and those outside the 1-hour catchment deemed to be the marginalized population.

| Subcounty | Total  population | Mean travel time | Population (within 30 minutes) | Population (within 1-hour) | Population (outside 1-hour) |
| --- | --- | --- | --- | --- | --- |
| Bumula | 221053 | 31 | 111214 (50.31%) | 218882 (99.02%) | 2171  (0.98%) |
| Bungoma Central | 179217 | 25 | 130527 (72.83%) | 179211 (99.99%) | 6  (0.01%) |
| Bungoma East | 120547 | 27 | 80322  (66.63%) | 120341 (99.83%) | 206  (0.17%) |
| Bungoma North | 132331 | 27 | 85618  (64.7%) | 130565 (98.67%) | 1766  (1.33%) |
| Bungoma South | 302844 | 27 | 224393  (74.1%) | 299905 (99.03%) | 2939  (0.97%) |
| Bungoma West | 131996 | 27 | 86669  (65.66%) | 130335 (98.74%) | 1661  (1.26%) |
| Cheptais | 115960 | 56 | 36514  (31.49%) | 83704  (72.18%) | 32256  (27.82%) |
| Kimilili | 172246 | 23 | 133315  (77.4%) | 172186 (99.97%) | 60  (0.03%) |
| Mt Elgon | 86572 | 32 | 39493  (45.62%) | 78134  (90.25%) | 8438  (9.75%) |
| Tongaren | 104646 | 58 | 11989  (11.46%) | 69629  (66.54%) | 35017  (33.46%) |
| Webuye West | 167488 | 28 | 116228 (69.39%) | 166952 (99.68%) | 536  (0.32%) |
| Bungoma County | **1734900** | **33** | **1056282 (60.9%)** | **1649844 (95.1%)** | **85056**  **(4.9%)** |

**Table S3**: Number of enumeration areas and population in each subcounty based on the accessibility levels (high, moderate and low) from the computed spatial accessibility index (SPAI).

|  | No. of EAs | | | Population  (%) | | |
| --- | --- | --- | --- | --- | --- | --- |
| Subcounty | **High** | **Moderate** | **Low** | **High** | **Moderate** | **Low** |
| Bumula | 23 | 87 | 83 | 29916 (13.53%) | 102291 (46.27%) | 88846 (40.19%) |
| Bungoma Central | 20 | 86 | 35 | 19573 (10.92%) | 114937 (64.13%) | 44707 (24.95%) |
| Bungoma East | 38 | 39 | 30 | 55957 (46.42%) | 36997 (30.69%) | 27593 (22.89%) |
| Bungoma North | - | 49 | 42 | - | 61343 (46.36%) | 70988 (53.64%) |
| Bungoma South | 79 | 55 | 48 | 156203 (51.58%) | 82577 (27.27%) | 64064 (21.15%) |
| Bungoma West | 24 | 67 | 32 | 32149 (24.36%) | 72262 (54.75%) | 27585  (20.90%) |
| Cheptais | 41 | 32 | 117 | 30160 (26.01%) | 20385 (17.58%) | 65415 (56.41%) |
| Kimilili-Bungoma | 42 | 46 | 19 | 60499 (35.12%) | 78289 (45.45%) | 33458 (19.42%) |
| Mt Elgon | 31 | 39 | 41 | 26949 (31.13%) | 26843 (31.01%) | 32780 (37.86%) |
| Tongaren | - | 7 | 71 | - | 7144  (6.83%) | 97502 (93.17%) |
| Webuye West | 44 | 50 | 26 | 72395 (43.22%) | 65905 (39.35%) | 29188 (17.43%) |
| Bungoma County | **342** | **557** | **544** | **483801 (27.89%)** | **668973 (38.56%)** | **582126 (33.55%)** |

### **References**

1. Ray N, Ebener S: AccessMod 3.0: computing geographic coverage and accessibility to health care services using anisotropic movement of patients. International Journal of Health Geographics. 2008; 7(1):63.

2. Macharia PM, Odera PA, Snow RW, Noor AM: Spatial models for the rational allocation of routinely distributed bed nets to public health facilities in Western Kenya. Malaria journal. 2017; 16(1):367.

3. Ouko JJO, Gachari MK, Sichangi AW, Alegana V: Geographic information system‐based evaluation of spatial accessibility to maternal health facilities in Siaya County, Kenya. Geographical Research. 2019; 57(3):286-298.

4. Ocholla IA, Agutu NO, Ouma PO, Gatungu D, Makokha FO, Gitaka J: Geographical accessibility in assessing bypassing behaviour for inpatient neonatal care, Bungoma County-Kenya. BMC pregnancy and childbirth. 2020; 20(1):1-16.

5. KNBS: Kenya Population and Housing Census Volume 1: Population by County and Sub-County. 2019.

6. Macharia PM, Giorgi E, Thuraniira PN, Joseph NK, Sartorius B, Snow RW, Okiro EA: Subnational variation and inequalities in under-five mortality in Kenya since 1965. BMC Public Health. 2019; 19(1):146.

7. KNBS: Comprehensive Poverty Report Children, Youth, Women, Men & The Elderly from national to county level. Kenya National Bureau of Statistics Nairobi, Kenya. 2020. <https://www.genderinkenya.org/wp-content/uploads/2020/08/CPR-Report-10_08_2020.pdf> Accessed 5 September 2022
